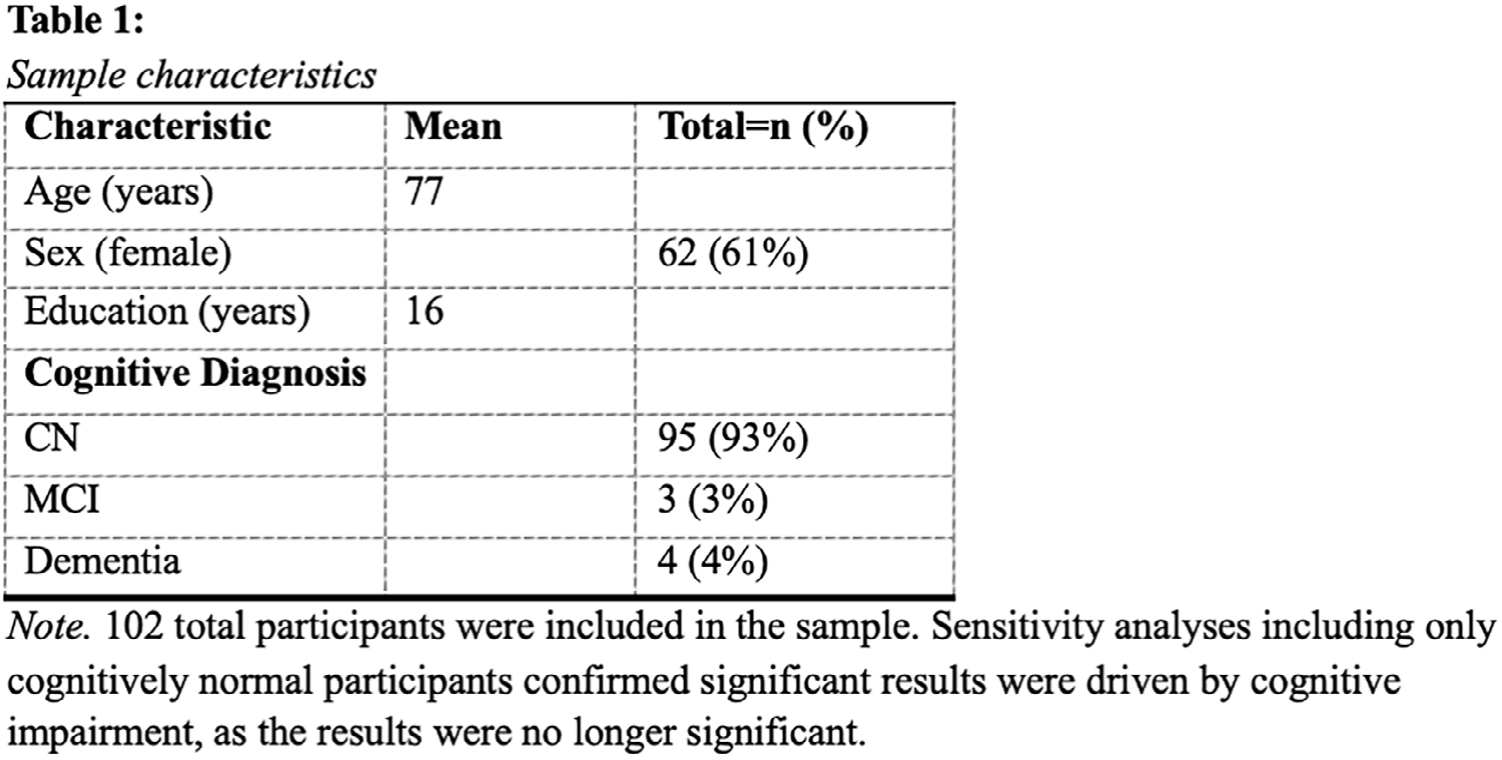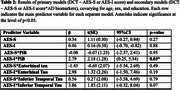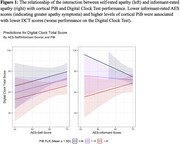# The interactive influence of cortical amyloid levels and apathy symptoms on Digital Clock Test performance

**DOI:** 10.1002/alz70861_108815

**Published:** 2025-12-23

**Authors:** Natalie R Scher, Phebe Palmer, Abigail LaCasse, Jessie Fanglu Fu, Talia L. Robinson, Dorene M. Rentz, Rebecca E. Amariglio, Kathryn V Papp, Yakeel T. Quiroz, Deborah Blacker, Reisa A. Sperling, Keith A. Johnson, Gad A. Marshall, Jennifer R. Gatchel, Catherine E Munro

**Affiliations:** ^1^ Massachusetts General Hospital/Brigham & Women's Hospital, Boston, MA USA; ^2^ Massachusetts General Hospital, Boston, MA USA; ^3^ Harvard Medical School, Boston, MA USA; ^4^ Brigham and Women's Hospital, Boston, MA USA; ^5^ William James College, Newton, MA USA; ^6^ Athinoula A Martinos Center for Biomedical Imaging, Massachusetts General Hospital, Harvard Medical School, Charlestown, MA USA; ^7^ Massachusetts General Hospital, Harvard Medical School, Boston, MA USA; ^8^ Center for Alzheimer Research and Treatment, Brigham and Women’s Hospital, Boston, MA USA; ^9^ Center for Alzheimer Research and Treatment, Brigham and Women's Hospital, Boston, MA USA; ^10^ Center for Alzheimer's Research and Treatment, Brigham and Women’s Hospital, Harvard Medical School, Boston, MA USA; ^11^ Boston University, Boston, MA USA; ^12^ Grupo de Neurociencias de Antioquia, University of Antioquia, Colombia, Medellín, Antioquia Colombia; ^13^ Harvard T.H. Chan School of Public Health, Boston, MA USA; ^14^ Center for Alzheimer Research and Treatment, Department of Neurology, Brigham and Women’s Hospital, Boston, MA USA; ^15^ Massachusetts General Hospital, Harvard Medical School, Department of Neurology, Boston, MA USA; ^16^ McLean Hospital, Belmont, MA USA; ^17^ Brigham and Women’s Hospital/Massachusetts General Hospital, Boston, MA USA

## Abstract

**Background:**

The Digital Clock Test (DCT) is a novel, digitized version of the Clock Drawing Test that is sensitive to Alzheimer’s disease (AD) pathological burden (i.e., amyloid, tau) and cognitive impairment, yet provides a wealth of additional data. However, it is unclear how apathy, a common neuropsychiatric symptom often associated with AD pathology and cognitive decline, affects performance on the DCT. This study investigated the impact of apathy, alone and in combination with AD‐biomarkers, on DCT performance in older adults.

**Methods:**

Participants (*n* =102; mean age=77; 61% female; mean years education=16, 7 cognitively‐impaired) were from the Harvard Aging Brain Study (Table 1). Participants completed the DCT, self‐ and informant‐rated versions of the Apathy Evaluation Scale (AES; lower scores=greater apathy), and the self‐rated Geriatric Depression Scale (GDS) concurrently, then underwent amyloid (PiB) and tau (FTP) PET imaging within 18‐months of clinical assessments. Separate, cross‐sectional models examined self‐ and informant‐rated AES scores as predictors of DCT scores. Models were repeated with interactions between apathy and AD biomarkers (i.e., cortical amyloid aggregate and inferior temporal & entorhinal cortex tau) as primary predictors. All models controlled for age, sex, and education. Sensitivity analyses were run removing cognitively‐impaired participants and examining GDS scores as a covariate.

**Results:**

AES scores did not predict DCT scores; however, the interaction between informant‐rated AES scores and PiB was predictive of DCT scores, such that individuals with higher PiB uptake and lower informant‐rated AES scores (indicating greater apathy) had lower DCT scores (Table 2; Figure 1). Results were unchanged when covarying for depression, but significance was lost when cognitively‐impaired participants were removed. Models with tau interactions were not significant, though the interaction with inferior temporal tau was trending.

**Conclusions:**

In this sample of older adults, apathy symptoms alone did not impact DCT performance, though the presence of informant‐rated apathy symptoms and elevated amyloid burden together contributed to worse performance on the DCT, particularly in cognitively‐impaired individuals. This supports the use of the DCT as an efficient screening tool and highlights the need to further investigate the potential synergistic relationship between amyloid and apathy that could contribute to worse cognitive outcomes.